# Occurrence and Risk Characterization of Ochratoxin A in Commercial Cow's Milk From Markazi Province, Iran

**DOI:** 10.1002/fsn3.72517

**Published:** 2026-10-26

**Authors:** Pedram Hashemi, Mohammadhosein Movassaghghazani, Milica Zekovic

**Affiliations:** ^1^ Faculty of Veterinary Medicine, Shab. C Islamic Azad University Shabestar Iran; ^2^ Department of Food Hygiene and Quality Control, Faculty of Veterinary Medicine, Shab. C Islamic Azad University Shabestar Iran; ^3^ Centre of Research Excellence in Nutrition and Metabolism, Institute for Medical Research, National Institute of Republic of Serbia University of Belgrade Belgrade Serbia

**Keywords:** margin of exposure, milk, ochratoxin A, risk assessment

## Abstract

Ochratoxin A (OTA) is a heat‐resistant mycotoxin with recognized nephrotoxic and carcinogenic effects. This study aimed to determine OTA occurrence in commercial milk marketed in Markazi Province, Iran, and to evaluate the associated health risks for consumers. A total of 180 cow's milk samples comprising raw (*n* = 60), pasteurized (*n* = 60; seven commercial brands), and UHT milk (*n* = 60; twelve commercial brands) were randomly collected across four seasons from retail outlets in Arak, Khomein, and Mahallat between October 2024 and 2025. All samples were analyzed using HPLC‐FLD. Dietary exposure was estimated for adults and children using the Estimated Daily Intake (EDI), and risk characterization was performed using the Margin of Exposure (MOE) approach. OTA was detected in 99.44% of samples, with an overall mean concentration of 57.99 ± 37.21 ng/kg. Raw milk showed the highest contamination levels, reaching 126.90 ng/kg in summer, whereas UHT milk exhibited the lowest concentrations (10.38 ng/kg in winter). Risk assessment revealed that MOE values for neoplastic effects in children consuming raw (4356) and pasteurized milk (7967) were below the safety threshold of 10,000, indicating potential carcinogenic concern. In contrast, MOE values for adults across all milk types remained within acceptable limit. The widespread detection of OTA suggests contamination within the regional feed supply chain. Given OTA's thermal stability, lower concentrations in industrially produced UHT milk likely reflect superior hygiene and quality‐control practices. These findings highlight the need for establishing national OTA limits in milk and improving feed storage and management to reduce exposure, particularly among children.

## Graphical abstract

This study evaluated Ochratoxin A (OTA) contamination in 180 commercial milk samples from Markazi province, Iran. OTA was detected in 99.44% of samples, with raw milk exhibiting the highest levels. Health risk assessment revealed that Margin of Exposure (MOE) values for children consuming raw and pasteurized milk fell below safe thresholds, indicating potential carcinogenic concerns.

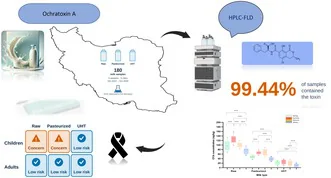

## 1 Introduction

Ochratoxin A (OTA), with the chemical formula C_20_H_18_ClNO_6_ and a molecular weight of 403.82 g/mol, is one of the most dangerous natural mycotoxins produced by several species of the *Aspergillus* genus (such as *A. ochraceus* , *A. carbonarius* , and *A. westerdijkiae*) and *Penicillium* (especially *P. verrucosum* ) (Schrenk et al. 2020). OTA is a white, odorless crystalline compound with low water solubility and moderate solubility in polar organic solvents. Its acid–base properties allow it to exist in non‐ionic, monoanionic, and dianionic forms depending on pH, and its strong fluorescence derived from the isocoumarin moiety makes it highly amenable to detection by fluorescence‐based chromatographic methods. These properties also explain why OTA remains detectable in dairy products and why sensitive analytical techniques are required for its quantification (Schrenk et al. 2020).

The occurrence of OTA in agricultural commodities is strongly influenced by environmental conditions, particularly temperature and humidity, which affect the growth of ochratoxigenic fungi and subsequent toxin production. *P. verrucosum* is the main contaminant in temperate and cold regions (such as Northern Europe and Canada), and *Aspergillus* species are responsible for contamination in warm and tropical regions (Siantar et al. 2008).

This toxin is widely found in grains, legumes, coffee, grapes, and their by‐products, and due to its pronounced thermal stability, OTA may persist throughout food‐processing procedures, thereby remaining a potential source of dietary exposure even in processed food products (Trucksess and Pohland 2008).

OTA has attracted considerable toxicological attention because of its nephrotoxic, immunotoxic, developmental, and potentially carcinogenic properties. Based on sufficient evidence of carcinogenicity in experimental animals and limited evidence in humans, the International Agency for Research on Cancer (IARC) has classified OTA as a Group 2B carcinogen (IARC 1993).

According to bioassay research, long‐term exposure to OTA has been associated with conditions such as Balkan Endemic Nephropathy (BEN) in humans, although current evidence indicates that BEN is a multifactorial disease. OTA exposure has also been shown to induce kidney and mammary gland tumors in experimental animal studies (Malir et al. 2016).

OTA has a very strong affinity for binding to plasma proteins (especially albumin) and has an extremely long elimination half‐life in the human body, increasing the risk of its bioaccumulation in tissues (Singh and Kumari 2022).

The presence of mycotoxins in dairy products reflects contamination of livestock feed. Ruminants possess a natural ability to detoxify OTA through microbial degradation in the rumen, converting the toxin into the less harmful metabolite ochratoxin‐α. Overall, the ability of cattle to degrade OTA is substantial but highly sensitive to physiological and nutritional factors. Any disruption in rumen function or exposure to heavily contaminated feed can reduce detoxification efficiency and increase the likelihood of OTA transfer into milk. Because of its prolonged half‐life, OTA can accumulate in the tissues of monogastric livestock such as pigs and may consequently enter meat and meat products. In contrast, ruminants exhibit efficient ruminal degradation of OTA to ochratoxin‐α, resulting in generally low concentrations in milk and edible tissues. These interspecies differences highlight the importance of rumen functionality and metabolic capacity in determining OTA carry‐over into animal‐derived foods (Battacone et al. 2010; Schrenk et al. 2020).

Studies suggest that because of the lack of fungicide use in feed production and differences in ration composition, the likelihood of OTA contamination in milk may be higher in organic farming systems than in industrial operations (Pattono et al. 2011; Schrenk et al. 2020).

The reported occurrence of OTA in milk varies considerably across geographic regions, ranging from non‐detectable levels in some European countries to substantially higher prevalence in regions with warmer climates and greater challenges in feed storage and quality control. For example, studies in Italy have reported prevalence rates of 20%–87% (Battacone et al. 2010).

In its latest assessment using the margin of exposure (MOE) approach, the European Food Safety Authority (EFSA) expressed concern about the genotoxic and tumorigenic hazards of this toxin associated with dietary exposure (Schrenk et al. 2020).

Despite the growing international literature on OTA contamination in dairy products, data from Iran remain limited, particularly with respect to seasonal variation and consumer risk assessment. Given the strategic position of Markazi Province in Iran's dairy industry and the region's climatic diversity, which can provide suitable conditions for toxin‐producing fungi, careful monitoring of this contaminant in the food chain is essential. This study was designed to (i) determine the occurrence and concentration of OTA in raw, pasteurized, and UHT cow's milk produced in Markazi Province, Iran; (ii) evaluate seasonal and product‐specific variations in contamination; and (iii) assess the potential health risks associated with OTA exposure using the Margin of Exposure (MOE) approach for both adults and children. By integrating analytical determination with risk characterization, the study provides the first comprehensive assessment of OTA contamination and carcinogenic risk in milk from this region.

## 2 Materials and Methods

### 2.1 Reagents

OTA was obtained from Sigma‐Aldrich (Darmstadt, Germany). Solvents with appropriate purity for high‐performance liquid chromatography with a fluorescence detector (HPLC‐FLD) were supplied by Merck (Germany). All chemicals used in this study were of chromatographic grade.

### 2.2 Samples

In total, 180 cow's milk samples were analyzed, categorized into three groups: raw (*n* = 60), pasteurized (*n* = 60, representing seven brands), and UHT (*n* = 60). These samples were randomly gathered from various retail markets in Arak, Khomein, and Mahallat, Markazi Province, throughout the four seasons between October 2024 and 2025 (Figure 1). The number of samples collected from each milk type (raw, pasteurized, and UHT) was kept constant across all four seasons to ensure uniform seasonal representation and comparability. All pasteurized and UHT milk samples were commercial products obtained from retail outlets. Pasteurized milk (produced by seven commercial brands) consisted exclusively of standard low‐fat formulations (2% fat), and no skimmed, lactose‐free, fortified, or flavored variants were included. UHT samples comprised only regular full‐fat milk (3.0% fat) manufactured by twelve commercial brands. Specialty formulations such as lactose‐free, high‐protein, vitamin‐fortified, or reduced‐fat UHT milk were intentionally excluded to prevent matrix variability that could affect extraction efficiency, chromatographic performance, or dietary exposure calculations. This sampling protocol was specifically structured to ensure a representative assessment of seasonal and regional influences on OTA levels. Upon purchase, samples were immediately placed in sterile vessels and frozen at −20°C to maintain integrity until further testing.

**FIGURE 1:**
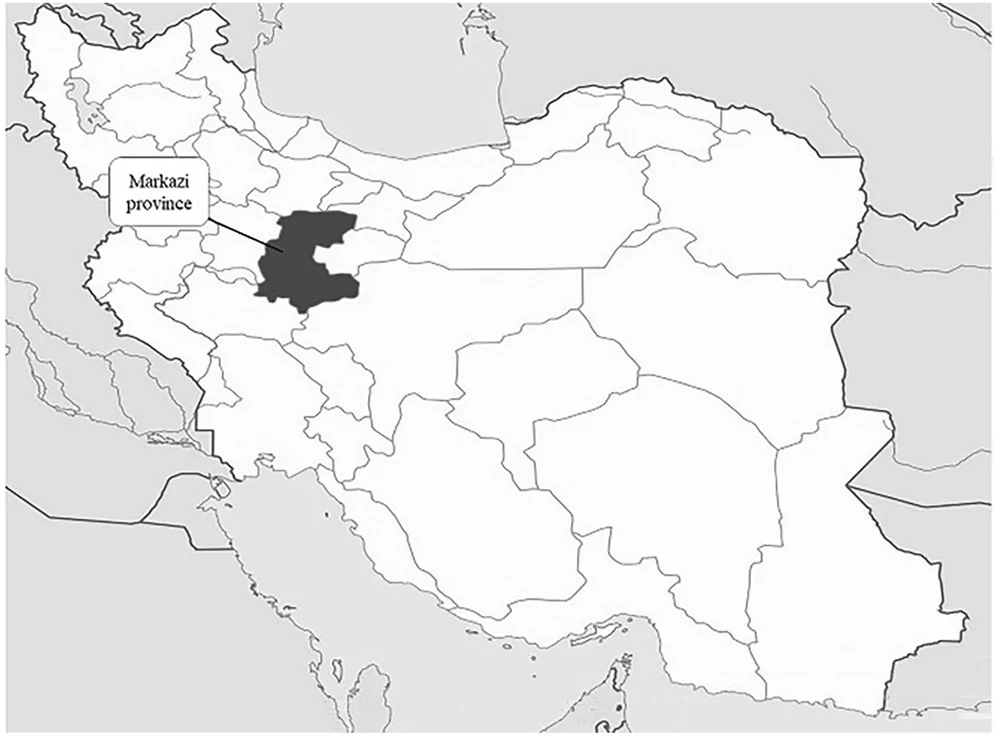
The map of Iran shows the Markazi Province, which was chosen for seasonal milk sampling.

### 2.3 Extraction of OTA in Milk Samples

To prevent a reduction in OTA levels due to salt formation, precipitation, or adsorption to glassware, all containers used were washed with methanol before starting the tests. For acidification, 2.5 mL of milk was mixed with 20 μL of 18% sulfuric acid (v/v) to reach a final pH of approximately 2.0 ± 0.5. To defat the sample, 10 mL of hexane was added, and the mixture was blended for 1 min. The sample was then centrifuged at room temperature at 4000 rpm for 15 min, and the hexane phase was discarded. Next, 5 mL of acetonitrile was added to the remaining aqueous phase and mixed horizontally at 600 rpm for 30 min. The resulting solution was filtered through cellulose filters with a pore size of 0.45 μm. The tube and filter were each rinsed twice with 1 mL of acetonitrile. Then, 1.5 mL of the resulting extract was evaporated to dryness at 60°C and reconstituted with 10 mL of distilled water (Pattono et al. 2011; Prado et al. 2003). For the cleanup step, 10 mL of the diluted extract was passed through an Ochratest immunoaffinity column (Vicam L.P., USA) at a flow rate of one drop per second. The column was initially washed with 5 mL of a solution containing 2.5% NaCl and 0.5% NaHCO_3_, followed by 5 mL of distilled water at a rate of 1–2 drops/s. Finally, OTA was eluted from the immunoaffinity column using 2 mL of methanol. The eluate was evaporated to dryness under a gentle nitrogen stream, and the residue was reconstituted with 300 μL of the mobile phase before chromatographic analysis (Pattono et al. 2011; Prado et al. 2003).

### 2.4 Chromatographic Conditions

The concentration of OTA was quantified using HPLC with a Unicam‐Crystal‐200 instrument (UK). HPLC‐FLD analysis was conducted using a Merck‐Hitachi L‐7100 pump equipped with a vacuum membrane degasser (model L‐7614; Merck, Darmstadt, Germany). Sample injection was performed via a Rheodyne 7725i injector valve with a 50 μL loop, and the column used was a Merck PuroSpher Star RP‐18 end‐capped with dimensions of 250 × 4 mm and a particle size of 5 μm. The fluorescence detector was a Merck‐Hitachi L‐7480 (Merck, Darmstadt, Germany) set at an excitation wavelength of 333 nm and an emission wavelength of 460 nm. The mobile phase consisted of a mixture of water, acetonitrile, and acetic acid in a volume ratio of 49.5:49.5:1, at a flow rate of 1.0 mL/min. The retention time (Rt) for OTA was determined to be 14.3 min.

### 2.5 Method Validation

The linearity of the method was confirmed using six calibration levels (0–40–80–120–160–200 ng/kg), prepared by diluting OTA stock solution in a toluene–acetic acid mixture (99:1, v/v). Each level was analyzed in triplicate, and the calibration curve showed excellent linearity (R^2^ = 0.9815) (Figure 2). Sensitivity was evaluated through the limit of detection (LOD) and limit of quantification (LOQ), defined by signal‐to‐noise ratios of 3:1 and 10:1, and also calculated using the equations LOD = 3.3*σ*/S and LOQ = 10σ/S, where σ represents the standard deviation of blank responses and S is the calibration slope.

**FIGURE 2:**
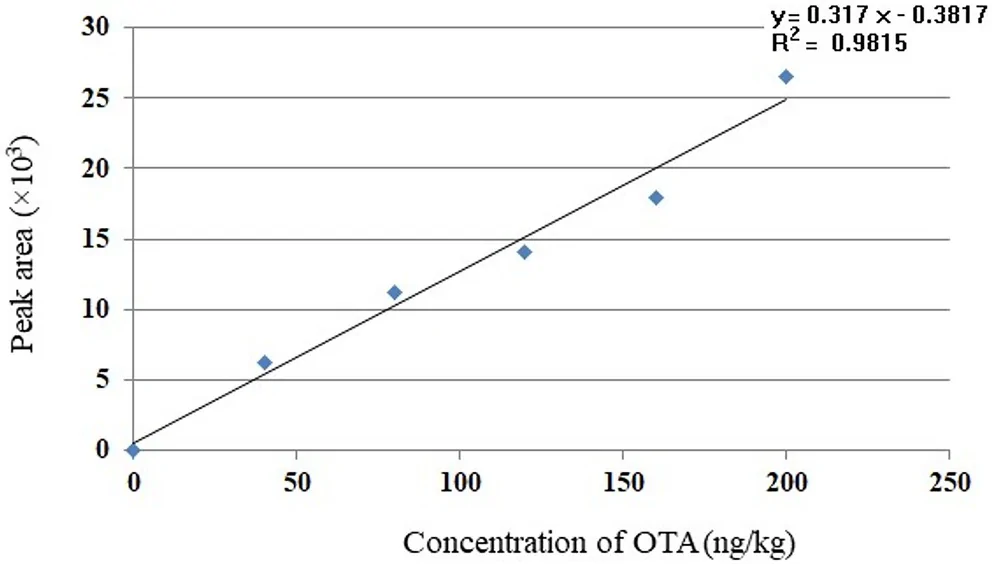
Calibration curve for the quantification of OTA by HPLC, demonstrating linearity with a coefficient of determination *r* ^2^ = 0.9815.

Specificity was verified by comparing chromatograms of blank samples with those fortified at 50 ng/kg, confirming the absence of interfering peaks at the OTA retention time. Matrix effects were assessed by calculating the signal suppression/enhancement (SSE) ratio, which compares the slopes of matrix‐matched calibration curves (bmm) to those prepared in solvent (bsol). This calculation is defined by the following Equation (1).

(1) $$\mathrm{SSE}\;\left(\%\right)=\frac{\mathrm{bmm}-\text{bsol}}{\text{bsol}}\times 100$$

Positive signal suppression/enhancement (SSE) values indicated signal enhancement, although high recovery rates demonstrated that matrix effects were minimal. No interfering signals were detected near the OTA retention time despite the complexity of the matrices. Method precision was determined through intra‐day and inter‐day repeatability at three spiking levels (40, 80, and 120 ng/kg), with results expressed as relative standard deviation (RSD).

### 2.6 Risk Assessment of OTA Exposure

For milk consumers, the estimated dietary intake (EDI) or dietary exposure was determined using Equation (2).

(2) $$\mathrm{EDI}\;\left(\mathrm{ng}/\mathrm{kg}\;\mathrm{bw}/\mathrm{day}\right)=\frac{\text{Mean of}\;\mathrm{OTA}\;\text{in milk}\times \text{Daily milk intake}}{\text{Average body weight}}$$

Mean of OTA in milk (ng/kg).

Daily milk intake (kg/day).

Average body weight (kg).

According to Behtarin and Movassaghghazani's study, the average daily milk consumption was 0.192 kg per person. For exposure assessment, a body weight of 70 kg for adults and 15 kg for children was assumed (Behtarin and Movassaghghazani 2024).

#### 2.6.1 Margin of Exposure

The Margin of Exposure (MOE) approach was applied to estimate the potential health risks associated with OTA intake from different milk types consumed in Markazi Province. MOE was calculated as the ratio of the benchmark dose lower confidence limit for a 10% response (BMDL_10_) to the estimated daily intake (EDI), as expressed in Equation (3).

(3) $$\mathrm{MOE}=\frac{\mathrm{BMD}L_{10}}{\mathrm{EDI}}$$

#### 2.6.2 Non‐Neoplastic Benchmark Dose (BMDL_10_ )

For assessing non‐carcinogenic health effects, the benchmark dose lower confidence limit associated with a 10% adverse response (BMDL_10_) was used as the toxicological reference value. The European Food Safety Authority (EFSA) established a BMDL_10_ of 4.73 μg/kg bw/day, derived from renal damage observed in pigs, an animal model known for its high susceptibility to OTA‐induced kidney toxicity. This value forms the basis for MOE calculations in evaluating non‐neoplastic risks (Schrenk et al. 2020).

#### 2.6.3 Neoplastic Benchmark Dose (BMDL_10_ )

To evaluate the carcinogenic potential of OTA, EFSA identified a separate BMDL_10_ of 14.5 μg/kg bw/day, based on kidney tumor incidence in rat studies. Due to unresolved uncertainties regarding OTA's genotoxic mechanism, whether it acts through direct DNA interaction or indirect pathways, EFSA concluded that establishing a health‐based guidance value such as a tolerable daily intake (TDI) was not scientifically justified. Therefore, the MOE framework was recommended as the most appropriate method for characterizing neoplastic risks associated with OTA exposure (Schrenk et al. 2020).

### 2.7 Statistical Analysis

The Shapiro–Wilk test with a significance level of 0.05 was used to assess the normality of the data. Statistical analyses were performed using SPSS software (version 27) and R software (version 4.5.1), and graphical outputs were generated using GraphPad Prism (version 10.2.0). Given that the experimental design included two independent factors (“season” and “milk type”), a two‐way ANOVA was applied to evaluate both main effects and their interaction. The Shapiro–Wilk test confirmed that the OTA data were normally distributed; therefore, no data transformation or non‐parametric alternatives were required. For samples in which OTA concentrations were below the limit of detection, a value of zero was assigned for statistical analysis.

## 3 Results and Discussion

Table 1 summarizes the precision, accuracy, matrix effect, and sensitivity of the analytical method employed to determine OTA in milk samples. With RSD values less than 6.2%, the technique showed adequate intra‐day and inter‐day precision across all milk types (raw, pasteurized, and UHT). In particular, inter‐day RSDs varied between 2.95% and 6.10% while intra‐day RSDs ranged between 3.06% and 5.28%, showing acceptable repeatability and intermediate accuracy in line with well‐recognized validation requirements.

**TABLE 1:** Validation parameters for determining OTA in milk samples by the HPLC method.

Milk samples	Spiked level (ng/kg)	Intra‐day (*n* = 3) RSD^a^(%)	Inter‐day (*n* = 4) RSD^a^(%)	ER^b^ (%)	Matrix effect‐SSE^c^ (%)	LOD^d^ (ng/kg)	LOQ^e^ (ng/kg)
Raw	40	5.15	4.62	107.31	3.43	0.818	2.310
	80	3.33	2.95	96.54			
	120	4.29	3.51	91.70			
Pasteurized	40	3.72	5.79	88.25	4.14	0.321	1.616
	80	3.06	3.44	97.16			
	120	5.28	6.10	103.47			
UHT	40	4.19	4.69	92.72	2.51	0.493	3.114
	80	3.66	5.36	98.60			
	120	4.74	3.82	87.43			

^a^ Relative standard deviation for intra‐ and inter‐day precision at concentrations of 40,80, and 120 ng/kg of OTA.

^b^ Extraction recovery.

^c^ Signal suppression/enhancement – The SSE value reflects the average slope ratio across spiked levels for each matrix (raw, pasteurized, UHT).

^d^ Limit of detection (S/*N* = 3).

^e^ Limit of quantification (S/*N* = 10).

Additionally, the technique demonstrated good recovery rates (represented as extraction recovery, or ER), ranging from 87.43% to 107.31% at all spiking levels (40, 80, and 120 ng/kg). The accuracy of the approach for OTA quantification in complicated milk matrices is confirmed by these results, which fall within the permitted range of 70%–120%.

SSE was used to assess matrix effects; results ranged from 2.51% to 4.14%, indicating little influence from matrix components. Low LOD and LOQ, which varied depending on the kind of milk from 0.321 to 0.818 ng/kg and 1.616 to 3.114 ng/kg, respectively, validated the method's sensitivity. These results show that the technique is appropriate for identifying OTA at trace levels, which are well below legal limits.

Overall, the validation results show that the HPLC approach is reliable, sensitive, accurate, and precise for identifying OTA in different milk matrices, making it appropriate for regular monitoring and risk assessment.

OTA was detected in 179 of the 180 analyzed milk samples, corresponding to an overall detection frequency of 99.44%, indicating near‐ubiquitous contamination across the investigated dairy supply chain. The overall mean OTA concentration was 57.99 ± 37.21 ng/kg, ranging from 0.00 to 157.40 ng/kg.

Raw milk consistently showed the highest OTA levels, with seasonal means spanning from 73.98 ± 12.18 ng/kg in winter to 126.90 ± 16.66 ng/kg in summer. Raw milk consistently exhibited the highest OTA concentrations among the investigated milk categories, with seasonal mean values ranging from 73.98 ± 12.18 ng/kg in winter to 126.90 ± 16.66 ng/kg in summer. Pasteurized milk showed intermediate OTA concentrations, with seasonal averages varying between 38.22 ± 7.14 ng/kg and 66.51 ± 6.95 ng/kg during winter and summer, respectively. In contrast, UHT milk consistently displayed the lowest OTA concentrations, ranging from 10.38 ± 7.07 ng/kg in winter to 26.26 ± 9.00 ng/kg in summer.

A consistent seasonal pattern was observed across all milk categories, characterized by the highest OTA concentrations during summer and the lowest concentrations during winter. While the magnitude of contamination differed substantially among milk types, the direction of seasonal variation remained comparable throughout the study period.

Collectively, these results highlight the critical influence of milk origin, production scale, and supply‐chain hygiene on OTA contamination. From a public health perspective, the observed variability among milk categories underscores the importance of considering product‐specific contamination patterns when evaluating dietary exposure to OTA. In this regard, the consistently lower OTA concentrations observed in UHT milk throughout the study period may be relevant when interpreting potential differences in consumer exposure across milk categories.

Figure 3 presents the seasonal variations in OTA concentrations across raw, pasteurized, and UHT milk. Raw milk samples consistently displayed the highest OTA levels and variability across all seasons, followed by pasteurized milk, while UHT milk showed the lowest concentrations with minimal seasonal fluctuations.

**FIGURE 3:**
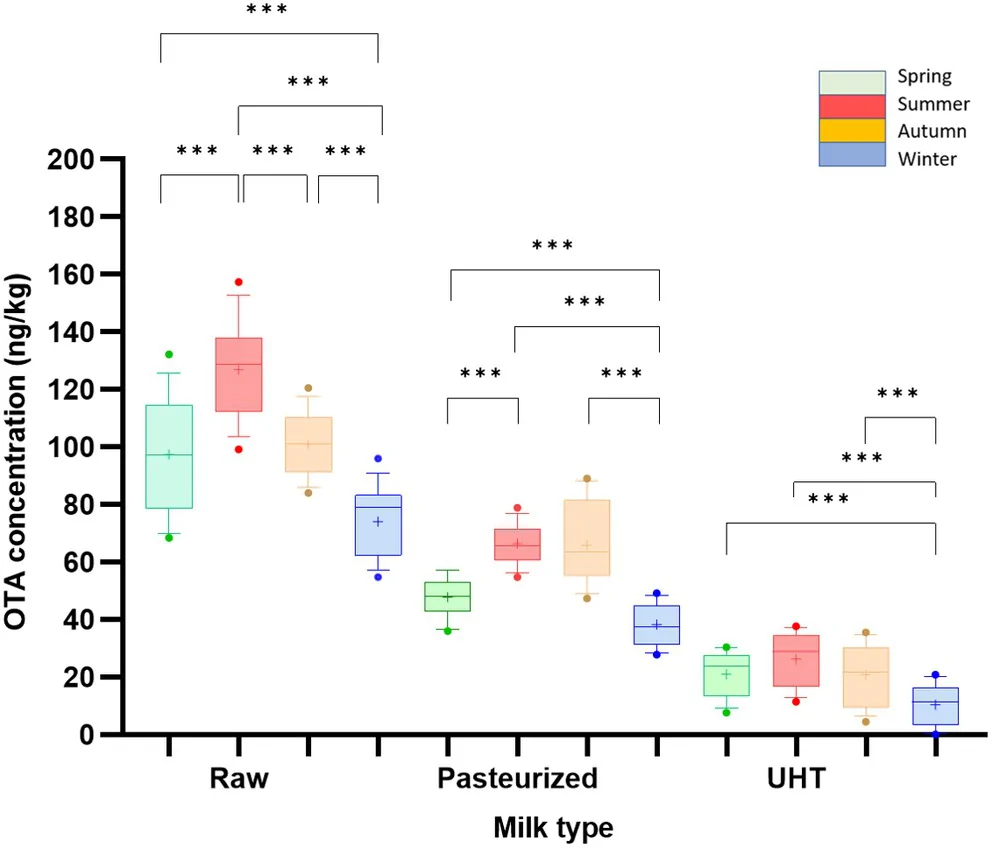
Seasonal variation in OTA concentrations across different milk types in Markazi Province, Iran. Boxes represent the 25th, 50th (median), and 75th percentiles; whiskers indicate the 10th and 90th percentiles. The central plus sign (+) denotes the mean. Brackets indicate statistically significant differences among seasons within each milk category, with asterisks denoting the corresponding significance level (***p < 0.001), as determined by the two‐way ANOVA interaction effect followed by appropriate pairwise comparisons.

Figure 4 illustrates the interaction between milk type and season on OTA concentration. OTA contamination was significantly higher in raw milk samples, particularly during summer, while UHT milk exhibited the lowest levels across all seasons. Two‐Way ANOVA revealed a significant interaction between milk type and season (F = 8.862, *p* < 0.001), indicating that seasonal variations in OTA levels differed among milk types. Raw milk showed a pronounced seasonal pattern, with the highest OTA concentrations in summer and the lowest in winter. Pasteurized milk showed moderate seasonal fluctuations, whereas UHT milk showed minimal seasonal change and consistently low OTA levels. These results confirm that the effect of season on OTA contamination is strongly dependent on milk type.

**FIGURE 4:**
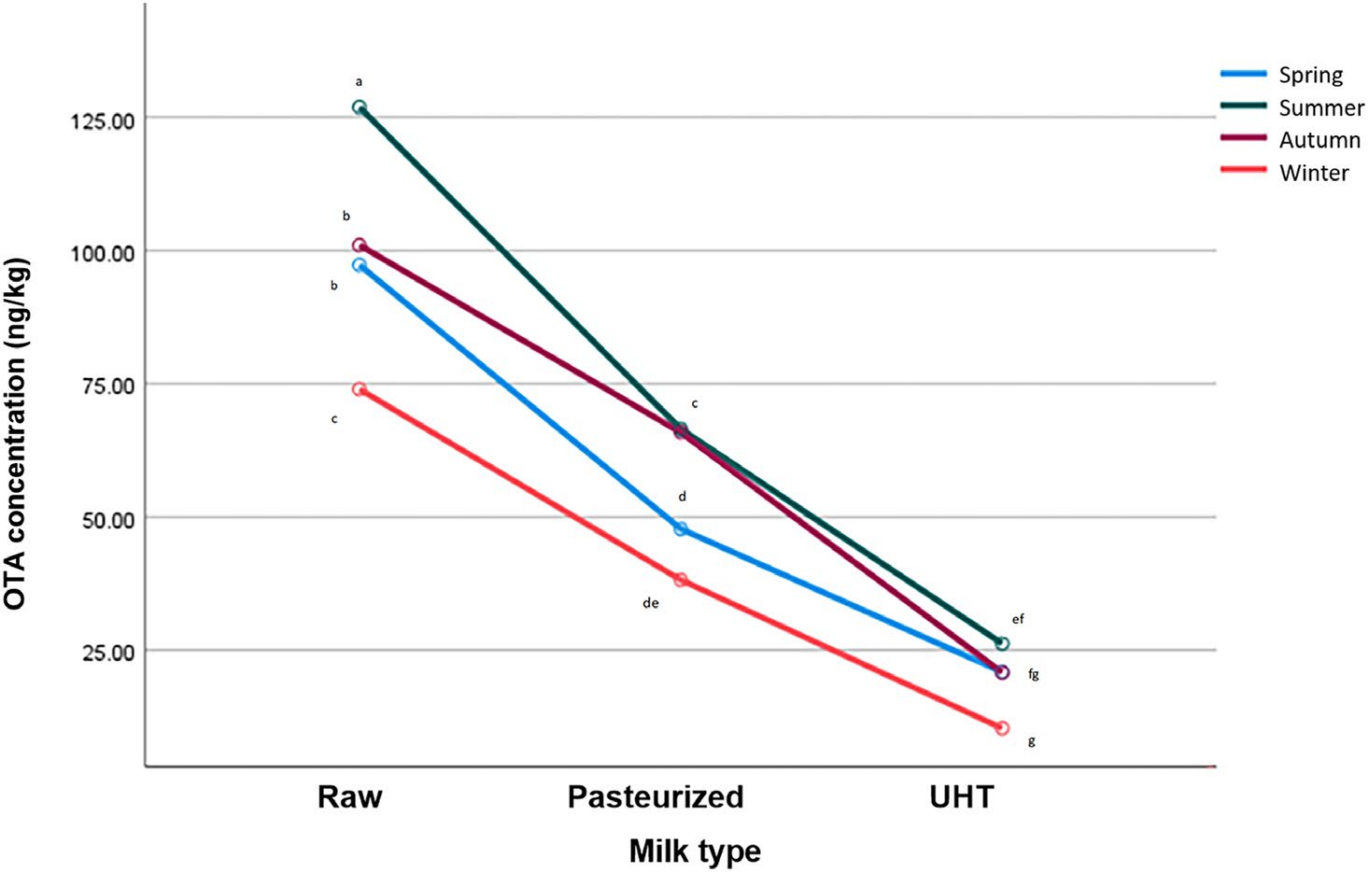
Interaction effect between milk type and season on the mean concentration of OTA in commercial cow's milk samples from Markazi Province, Iran. Different lowercase letters indicate statistically significant differences among means (*p* < 0.001).

Figure 5 presents the distribution of OTA concentrations according to milk type. A clear gradient was observed across categories, with OTA concentrations decreasing from raw to pasteurized and subsequently to UHT milk. Pairwise comparisons demonstrated statistically significant differences between milk categories (*p* < 0.001). In addition, the comparatively lower OTA concentrations observed in UHT milk may partly reflect a dilution effect, as this product is typically sourced from a larger number of supplying farms, resulting in greater blending of raw milk before processing.

**FIGURE 5:**
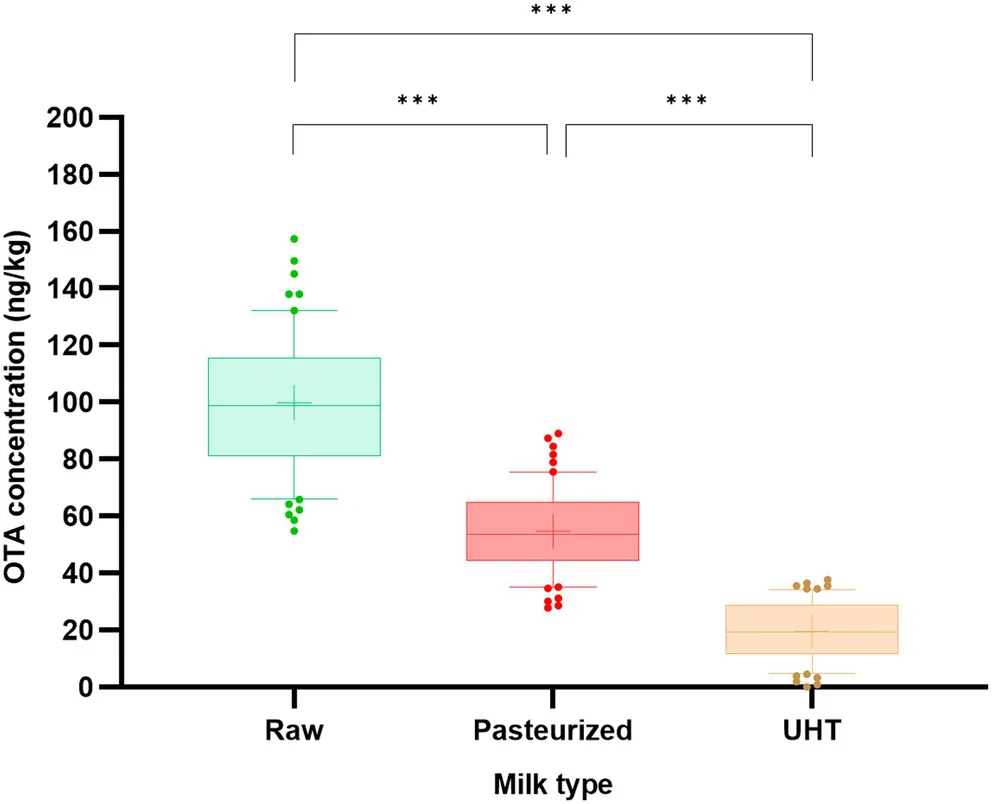
Variation of OTA concentrations across different milk types in Markazi Province, Iran. Boxes represent the 25th, 50th (median), and 75th percentiles; whiskers indicate the 10th and 90th percentiles. The central plus sign (+) denotes the mean. Significant differences in OTA concentrations among the milk types are indicated by asterisks (***p < 0.001), based on the main effects identified by the two‐way ANOVA.

Table 2 summarizes the EDI and MOE values for both non‐neoplastic and neoplastic effects associated with OTA exposure through consumption of raw, pasteurized, and UHT milk.

**TABLE 2:** EDI and MOE values for non‐neoplastic and neoplastic effects of OTA via consumption of raw, pasteurized, and UHT cow's milk in Markazi Province, Iran.

Milk type	Raw	Pasteurized	UHT
Mean daily consumption (kg)	0.192	0.192	0.192
EDI mean (ng OTA/kg bw/day) for adults	0.274	0.150	0.054
EDI mean (ng OTA/kg bw/day) for children	3.33	1.82	0.653
MOE values for adults (bw 70 kg)‐Non‐neoplastic	17,266	31,533	87,593
MOE values for children (bw 15 kg)‐Non‐neoplastic	1420	2599	7240
MOE values for adults (bw 70 kg)‐Neoplastic	52,920	96,667	268,519
MOE values for children (bw 15 kg)‐Neoplastic	4356	7967	22,200

*Note:* MOE > 200 (for non‐neoplastic effects): Indicates low health concern. MOE > 10,000 (for neoplastic effects): Indicates low health concern, assuming a direct genotoxic mode of action. MOE < threshold values: May indicate potential health concern, especially for vulnerable populations such as children.

Abbreviation: bw, body weight.

The potential health risks associated with dietary exposure to OTA from raw, pasteurized, and UHT milk were evaluated using the MOE approach. MOE values were calculated based on the EDI and the BMDL_10_ established by EFSA for both non‐neoplastic and neoplastic endpoints. The BMDL_10_ values applied were 4.73 μg/kg bw/day for non‐neoplastic effects and 14.5 μg/kg bw/day for neoplastic effects.

Daily OTA intake was estimated using the mean OTA concentrations in each milk type and age‐specific consumption patterns. For adults (70 kg body weight; 0.192 kg/day milk intake), the mean EDI values were 0.274, 0.150, and 0.054 ng/kg bw/day for raw, pasteurized, and UHT milk, respectively. For children (15 kg body weight; 0.5 kg/day milk intake), the corresponding EDI values were 3.33, 1.82, and 0.653 ng/kg bw/day. To improve the interpretability of our exposure assessment, we have incorporated a comparative analysis of the Estimated Daily Intake (EDI) values across different milk categories. By explicitly noting that the estimated OTA intake in children is approximately five‐fold higher for raw milk compared to UHT milk, we provide a clearer perspective on the magnitude of the observed exposure gradient. This addition not only clarifies the disparity in intake levels but also strengthens the logical transition from our exposure assessment data to the subsequent MOE‐based risk characterization.

The resulting MOE values for adults exceeded EFSA's safety thresholds for both non‐neoplastic (MOE > 200) and neoplastic (MOE > 10,000) outcomes across all milk types, indicating no appreciable health concern for this population group. In contrast, children exhibited substantially lower MOE values. For raw milk, the MOE for non‐neoplastic effects was 1420, and for neoplastic effects 4356, the latter falling below the 10,000 benchmark and suggesting a potential carcinogenic concern. Pasteurized milk also produced a neoplastic MOE below the threshold (7967), indicating moderate risk. UHT milk, however, yielded MOE values of 7240 (non‐neoplastic) and 22,200 (neoplastic), both comfortably above the corresponding safety criteria.

Overall, the results indicate that OTA exposure through milk consumption varies substantially according to both milk category and consumer age group. While the calculated MOE values suggest a low level of concern for adults across all investigated milk types, lower MOE values were observed in children, primarily due to their higher milk consumption relative to body weight. Taken together, these findings emphasize the importance of integrating both age‐specific consumption patterns and product‐specific contamination profiles into OTA exposure assessments. They further illustrate how differences in contamination burden among commonly consumed dairy products may translate into substantially different exposure scenarios, with potentially disproportionate implications for vulnerable population groups such as children.

The present study provides compelling evidence that OTA contamination constitutes a pervasive feature of the dairy supply chain in Markazi Province, Iran. The exceptionally high prevalence observed, together with the marked differences in contamination levels across milk categories and seasons, underscores the complexity of factors governing OTA occurrence in dairy products. Beyond the near‐ubiquitous detection of the toxin, particularly noteworthy was the persistence of a clear contamination gradient across all seasons, with concentrations decreasing from raw to pasteurized and subsequently to UHT milk, alongside a pronounced seasonal pattern characterized by elevated concentrations during summer. In addition, the comparatively lower OTA concentrations observed in UHT milk may partly reflect a dilution effect, as this product is typically sourced from a larger number of supplying farms, resulting in greater blending of raw milk before processing. These findings not only expand the currently limited body of evidence regarding OTA occurrence in milk within the region but also provide valuable insight into the factors shaping contamination patterns in dairy products and highlight the importance of considering production practices, supply‐chain characteristics, and temporal variability when assessing dietary exposure to this mycotoxin.

In recent years, growing attention has been directed toward mycotoxin surveillance throughout the Iranian food chain, reflecting increasing awareness of the potential health implications associated with chronic dietary exposure. Within this context, the presented data provide a valuable basis for future surveillance activities and may support ongoing efforts to strengthen exposure assessment and food‐safety monitoring frameworks for mycotoxins in Iran.

The contamination levels observed in the present study should be interpreted within the context of the considerable geographical variability reported worldwide, where OTA occurrence in milk has been shown to differ substantially across regions, production systems, and climatic conditions.

Given the wide variability in OTA contamination across regions and production systems, the occurrence data from previous studies have been consolidated into Table 3.

**TABLE 3:** Comparative summary of OTA occurrence in milk reported in previous studies.

Country/Region	Sample type	Sample size	Analytical method	OTA Occurrence (%)	Mean/Range (ng/L or ng/kg)	Risk assessment	Study/Year
East Azerbaijan, Iran	Pasteurized milk	31	HPLC	9.7%	NR/30–48 ng/kg	NR	(Badali et al. 2025)
Italy	Organic & conventional milk	63 (Organic milk)/20 (Conventional milk)	HPLC	Organic: 4.8%; Conventional: 0%	Organic: NR/70–110 ng/kg Conventional: ND	Total Weekly Intake (TWI) of OTA was nearly 1.52 ng/kg b.w.	(Pattono et al. 2011)
Norway	Organic & conventional milk	47 (Organic milk)/40 (Conventional milk)	HPLC	Organic: 10.6%; Conventional: 15%	Organic: NR/15–28 ng/kg Conventional: NR/11–58	OTA levels in milk were sufficient to exceed the EU TDI (5 ng/kg bw/day) in high‐milk‐consuming children.	(Skaug 1999)
Sudan	Raw milk	Milk: 20%	HPLC	Milk: 20%	Milk: 2730 ng/L	NR	(Elzupir et al. 2009)
Turkey	Bulk tank raw milk	40	ELISA	100%	2–270 ng/L	OTA levels in milk (15% occurrence) were high enough to exceed the EU TDI (5 ng/kg bw/day) for high‐milk‐consuming children.	(Keyvan et al. 2018)
Egypt	Raw milk	10	HPLC	70%	5134 ng/kg/13000–340 ng/kg	NR	(Younis et al. 2016)
Markazi Province, Iran	Raw, Pasteurized, UHT milk	180	HPLC‐FLD	99.44%	57.99 ± 37.21/0.00–157.40 ng/kg	Adults: MOE values for all milk types were above EFSA thresholds (no health concern). Children: Raw and pasteurized milk showed MOE values below the neoplastic benchmark, indicating potential risk; UHT milk remained within safe limits.	Present study

Abbreviations: ND, not detected; NR, not reported.

A review of previous studies demonstrates substantial regional variability in OTA contamination and associated health risks in milk. OTA occurrence has been reported at relatively low levels in East Azerbaijan (9.7%), Italy (4.8% in organic and 0% in conventional milk), and Norway (10%–15%), whereas markedly higher contamination has been documented in Turkey (100%) and Egypt (70%). Mean OTA concentrations also vary widely, reaching extremely high values in Sudan (2730 ng/kg) and Egypt (over 5000 ng/kg), far exceeding those observed in the present study. In comparison, milk samples from Markazi Province exhibited one of the highest occurrence rates reported to date (99.44%), although the mean concentration (57.99 ng/kg) remained moderate relative to high‐contamination regions. Risk assessments from previous studies similarly indicate that regions with elevated OTA levels such as Turkey, Sudan, and Egypt pose significant exposure concerns, particularly for children. In the present study, despite moderate OTA concentrations, the near‐universal contamination of milk resulted in lower MOE values for children consuming raw and pasteurized milk, highlighting a distinct public‐health concern driven primarily by widespread occurrence rather than concentration intensity. Overall, these comparisons underscore that OTA contamination in Markazi Province is both pervasive and epidemiologically relevant, necessitating improved feed management and monitoring strategies.

The lower OTA levels observed in UHT milk are unlikely to result from thermal processing, given the heat stability of the toxin. Instead, these reduced concentrations are more plausibly linked to the stringent hygienic standards and industrial‐scale sourcing typical of UHT production in Iran. Large dairy operations supplying UHT milk generally implement rigorous feed management, controlled storage, and systematic quality assurance, thereby minimizing fungal contamination at the farm level.

## 4 Conclusion

The present study demonstrated that OTA is a pervasive contaminant in the milk supply chain of Markazi Province, with an overall prevalence of 99.44%. Contamination levels were highest in raw milk, while pasteurized and UHT products showed substantially lower concentrations, suggesting that upstream sourcing practices and contamination‐control measures play a critical role in determining the final OTA burden. The persistence of OTA throughout conventional processing highlights the toxin's thermal stability and underscores the importance of preventive actions at the farm and feed‐management levels.

The exposure assessment revealed that children are more vulnerable to OTA, with MOE values for neoplastic endpoints in raw and pasteurized milk falling below the EFSA reference threshold of 10,000. Seasonal variation, particularly elevated concentrations during summer, further indicates that environmental and production‐related factors influence contamination dynamics.

Taken together, these findings emphasize the need for continuous surveillance of OTA in milk and animal feed, improved feed‐storage and handling practices, and targeted interventions during high‐risk periods. Importantly, in the absence of an established Iranian regulatory limit for OTA in milk, the results of this study support consideration of a provisional threshold of 50 ng/kg, a value aligned with international risk‐assessment benchmarks and consistent with the contamination profile observed in the region. Establishing such an evidence‐based preliminary limit could guide future legislative efforts and contribute to a more robust national framework for mycotoxin control.

Future research should focus on identifying critical control points for OTA reduction along the dairy supply chain, evaluating regional and seasonal drivers of contamination, and assessing the effectiveness of emerging mitigation strategies. Broader nationwide monitoring programs are also recommended to support the development of harmonized regulatory standards for OTA in milk.

## Author Contributions

**Pedram Hashemi:** investigation, data curation. **Mohammadhosein Movassaghghazani:** investigation, data curation, methodology, supervision, writing – original draft, writing – review and editing, project administration. **Milica Zekovic:** writing – review and editing.

## Conflicts of Interest

The authors declare no conflicts of interest.

## Data Availability

Data will be made available on request.
